# Relationship between Antibody Levels, IgG Binding to* Plasmodium falciparum*-Infected Erythrocytes, and Disease Outcome in Hospitalized Urban Malaria Patients from Dakar, Sénégal

**DOI:** 10.1155/2016/5381956

**Published:** 2016-08-03

**Authors:** Babacar Mbengue, Mouhamadou Mansour Fall, Maguette Sylla Niang, Birahim Niang, Marie Louise Varela, Antoine Marie Diatta, Moustapha Mbow, Kantome Ndiaye, Rokhaya Ndiaye Diallo, Alioune Dieye, Ronald Perraut

**Affiliations:** ^1^Service d'Immunologie FMPO, Université Cheikh Anta Diop de Dakar, Dakar, Senegal; ^2^Unité d'Immunogénétique, Institut Pasteur de Dakar, Dakar, Senegal; ^3^Service de Réanimation, Hôpital Principal de Dakar, Dakar, Senegal; ^4^Unité d'Immunologie, Institut Pasteur de Dakar, Dakar, Senegal

## Abstract

*Background*. Management of clinical malaria requires the development of reliable diagnostic methods and efficient biomarkers for follow-up of patients. Protection is partly based on IgG responses to parasite antigens exposed at the surface of infected erythrocytes (iRBCs). These IgG responses appeared low during clinical infection, particularly in severe disease.* Methods*. We analyzed the IgG binding capacity to the surface of live erythrocytes infected by knob positive FCR3 strain. Sera from 69 cerebral malaria (CM) and 72 mild malaria (MM) cases were analyzed by ELISA for IgG responses to five antigens from iRBC and by flow cytometry for IgG binding as expressed in labeling index ratio (LIR). The relationship between IgG levels, LIR, parasitemia, age, and the clinical outcomes was evaluated.* Results*. We found a significant decrease of LIR in adult CM fatal cases compared to surviving patients (*p* = 0.019). In MM, LIRs were correlated to IgG anti-iRBC and anti-*Pf*EMP3/5 levels. In CM, no correlation was found between LIR, IgG levels, and parasitemia.* Conclusion*. The IgG binding assay was able to discriminate outcome of cerebral malaria cases and it deserves further development as a potential functional-associated assay for symptomatic malaria analysis.

## 1. Introduction

The robust progress in integrated intervention strategies including artemisinin-based combination therapy (ACT), coverage with long-lasting insecticide-impregnated bed nets (LLINs), and systematic diagnosis using rapid tests (RDTs) has considerably reduced the burden of malaria in many countries. However, malaria still remains one of the major infectious diseases in tropical and subtropical regions, causing over 225 million clinical cases and around 600,000 deaths per year [1]. However, efforts are threatened by increasing resistance of parasite and* Anopheles* vectors to drug and insecticides, respectively [2–4]. The control and elimination of malaria require the development of efficient vaccines and of reliable diagnostic and follow-up methods to prevent early individual infection by* Plasmodium falciparum* species which is responsible for most of the deaths in human malaria. It causes severe cases such as cerebral malaria during which the parasites are sequestrated in brain through a complex mechanism that involves interactions between human adhesion molecules and parasitic Var proteins that are highly polymorphic [5]. Parasite sequestration results in brain microvasculature impairment, increased blood volume, and possible occlusion of brain microvessels [6, 7]. Cytokines and parasite toxins have also been shown to cause direct damage to the blood-brain barrier [8].

*P. falciparum* virulence mainly relies on immune escape strategies that include the modification of the adhesion properties of infected erythrocytes to the vascular endothelium and the parasite's ability to undergo antigenic variation. The main antigenic ligands responsible for both cytoadherence and antigenic variation are members of the* P. falciparum* Erythrocyte Membrane Protein-1 (*Pf*EMP-1) family [6, 9]. There is evidence that protection against malaria is partly based on antibody responses to diverse parasite antigens [10] including proteins exposed at the surface of infected red blood cells (iRBCs). These antigens are immediate targets for protection-associated antibodies involved in opsonization and phagocytosis of infected erythrocytes [11]. In fact, the key role of antibodies (Abs) in protection against malaria was demonstrated decades ago by passive transfer assays in humans [12, 13]. Additionally, it is known that repeated exposure to* P. falciparum* infection confers a partial immunity to malaria through mechanisms that involve the progressive acquisition of a panel of antibodies that recognize varieties of surface antigens from diverse isolates [14].

There is some evidence that protection against parasite infection is partly based on antibody responses to diverse parasite antigens [10] including those exposed at the surface of infected red blood cells (iRBCs). They are the first line of target antigens accessible for protection-associated antibodies involved in opsonization and immune phagocytosis of infected erythrocytes [11]. The variability of iRBC surface antigens (Ags) upon immune pressure complicates the evaluation of their potential role in controlling parasite density* in vivo*.

Previous studies showed that flow cytometry could measure the ability of Abs to bind to native parasite surface-associated iRBCs Ags [15, 16]. Their potential contribution to immune protection in rural endemic settings [17] and in postvaccinated individuals has been reported [18].

In this study, we further investigated the potential protection-associated role of IgG against iRBCs Ags in urban symptomatic patients hospitalized for mild and cerebral Malaria. We thus evaluated the relationship between IgG binding capacity and Ab responses to* P. falciparum* iRBCs-associated Ags measured by enzyme-linked immunosorbent assay (ELISA) in the context of the bioclinical symptoms from patients hospitalized for confirmed clinical malaria infection. The Ags tested were whole parasite extracts from schizont and IRBC and recombinant IRBC-associated Ags R23,* Pf*EB200, and* Pf*EMP3/Clone5 described below.

## 2. Materials and Methods

### 2.1. Study Area and Epidemiologic Context

The study was performed in Dakar (Senegal), an urban area corresponding to a malaria hypoendemic setting with a low level of seasonal transmission. The transmission was estimated to be around 0.5 to 1 infecting bite/person/year and occurred during the rainy season from September to December [19, 20]. The main malaria vector described was* Anopheles arabiensis*, and* P. falciparum* was the most widespread species accounting for 98% of cases [21]. Previous studies in this area revealed that malaria affected all age groups with the highest prevalence occurring in children. A mean incidence of 2.4% of clinical disease has been observed, with no difference between adults and children [21, 22].

### 2.2. Study Population, Ethical Statements, and Procedures

The study was performed at the Principal Hospital of Dakar. Patients were recruited every year during the rainy season from September to December in three successive years 1999, 2000, and 2001.

An informed consent was obtained from each participant and/or their relatives prior to inclusion, after giving them written or verbal information in their native language. The protocols were approved by the investigators' institutions, the National Ethical Committee and the Ministry of Health of Senegal.

Thin and thick blood smears were prepared from rapid diagnostic test (RDT) positive patients, in order to determine the parasite species and the level of parasitemia. Blood samples used in this study for immunological analysis were collected after determining the parasitological and clinical profiles of the patients. A questionnaire with clinical history and demographic information was recorded. Patients with malaria and any other coinfection were excluded as previously described [23]. Two categories of patients were enrolled: cerebral malaria (CM) and mild malaria (MM) patients.

The CM group consisted of 69 patients hospitalized for unarousable coma (nonpurposeful response or no response to a painful stimulus by Glasgow score < 9) with microscopically diagnosed* P. falciparum* infection and without other clinically evident cause of impaired consciousness such as hypoglycemia, meningitis, and encephalitis according to World Health Organization criteria [24]. Samples were taken at the admission before any treatment. All patients were managed by the same medical staff. The treatment protocol was based on the Senegalese national recommendations which are intramuscular quinine 20 mg/kg followed by 20 mg/kg every 8 h. Patients were examined every 4 h for the first 24 h and every 6 h thereafter. Fatal cases occurred during 1 to 4 days after admission. Surviving patients completely recovered after treatment. A total of 18 CM patients had a fatal outcome (FCM) while 51 subjects recovered with no sequelae (SCM).

Regarding MM, a total of 124 patients who were treated at the outpatient clinic of the hospital were initially enrolled. Of these, 72 patients had fever with* P. falciparum* parasitemia of <25000 parasites/*µ*L of blood, with no evidence of impaired consciousness or seizures at the time of enrolment. Based on previously reported criteria [23, 25], 52 patients were excluded. Blood samples from MM patients were obtained on the day of hospital admission.

### 2.3. Antigens

Two crude antigenic preparations were used. They consisted of (i) a lysate of* in vitro*-matured schizont-enriched* P. falciparum* from infected erythrocytes (*Pf*Schz) [26] and (ii) erythrocyte membranes from infected red blood cell (iRBCm). Membrane from nonparasitized erythrocyte (nRBCm) was also tested as negative controls. Erythrocyte membranes from iRBCm and nRBCm were prepared according to the method of Fairbanks et al. [27]. The total protein concentration of the parasite preparations was estimated by the Bio-Rad assay. All Ags were kept frozen at minus 80°C in working aliquots.

Three purified recombinant proteins fused to* Schistosoma japonicum* glutathione S-transferase (GST) in the pGEXA vector were used. R23 contains 11 copies of a 6-amino-acid repeat derived from the central domain of Ag R45, whose consensus sequence is HKSDS N/S/H [28].* Pf*EB200 contains 13 repeats with characteristic Glu-Glu dimers; it derives from* Pf*332, a conserved giant protein accessible on the surface of infected erythrocytes [29], in late schizont [30].* Pf*EMP3/5 (or* Pf*EMP3 clone 5) is a recombinant product expressing the 1,450-bp* Eco*RI fragment recovered from clone 5, isolated from the FUP/SP Palo Alto (alias FCR3) genomic expression library. It expresses the C-proximal region of* Pf*EMP3, a high-molecular weight host membrane-associated protein [31, 32]. The control protein was the GST carrier previously reported [23, 33].

### 2.4. ELISA Procedure

Antimalarial plasma IgG levels against schizont extracts* Pf*Schz, iRBCm, nRBCm, and purified recombinant proteins were determined using ELISA as described [26, 32]. Crude Ag preparation (15 *µ*g/mL) and recombinant proteins (1 *µ*g/mL) were coated on MaxiSorp® (Nunc®, Roskilde, Denmark) and Immulon-4® (Dynatech®, Springfield, VA, USA) plates respectively, and incubated overnight at 4°C. Coated plates were washed with PBS containing 0.5% Tween 20 and then blocked with PBS-5% bovine serum albumin (BSA) (Sigma® Chemicals St. Louis, MO, USA) for 1 h at 37°C. Plasma samples diluted 1/200 in PBS-1% BSA-0.5% Tween 20 were added and incubated for 2 h at 37°C. Peroxidase-conjugated polyclonal goat anti-human IgG [IgG*γ*-chain specific] (Cappel®, Organon Teknika, PA, USA) was added to the wells and the plates were incubated for 2 h at 37°C. Bound antibodies were revealed using a citrate buffer (pH 4) containing 160 *µ*g/L orthotoluidine and 10% H_2_O_2_. The reaction was stopped with 4 N H_2_SO_4_, and the optical densities (OD) were measured at 450 nm in a plate reader (Tecan® GmbH, Salzburg, Austria). Negative controls consisted of a pool of nonimmune European sera and a pool of naïve African sera. The positive control was a pool of sera from clinically immune adults living in Dielmo. Results were expressed as OD ratios: OD_serum sample_/OD_negative control_. The OD signals of the samples were individually corrected for the GST or nRBCm signal. Serum samples with OD ratios greater than 2 (which is over the OD of naïve control + 3 SD) were considered seropositive [26, 32, 33].

### 2.5. Flow Cytometry Technique for the Detection of IgG Binding to Live iRBC

To test for binding of plasma IgG to live iRBC, we used a double staining cytofluorimetric technique as previously described [15, 16]. A knob positive (K^+^) Uganda Palo Alto FUP strain of* P. falciparum* (alias FCR3) maintained in culture was used as a source of live iRBC. This strain was cultured in complete medium (RPMI 1640, 25 nM HEPES, 2.4 mM L-glutamine, 50 *µ*g/mL gentamicin, and 0.5% w/v Albumax). All these reagents were from Sigma Chemicals (St. Louis, MO, USA). The culture was maintained at 37°C in an atmosphere of 5% CO_2_, with daily changes of medium at 5% hematocrit and dilution with red blood cells when the parasitaemia exceeded 5% [34]. K^+^ iRBCs were continuously selected after plasmagel flotation at parasitemia about 5% and washed once in phosphate buffered saline (PBS) (Sigma Chemicals, St. Louis, MO, USA).

**Figure 1:**
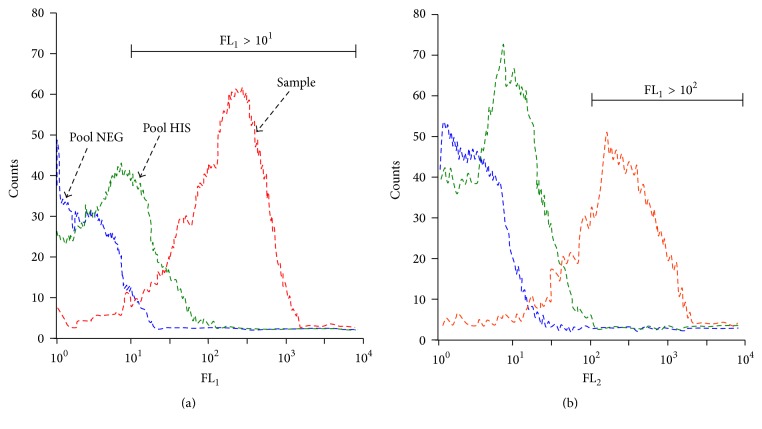
Examples of histograms of flow cytometry data acquisition. The two histograms show results from the acquisition of 5,000 events of a 3%* P. falciparum* culture after gating on FL_1_ > 10^1^ (a) and on FL_1_ > 10^2^ (b). The gating on high FL_1_ values (>10^2^) selected the mature population of the cultured parasites. The binding of human IgG to IRBC was revealed by an anti-IgG conjugated to thiazole orange (TO). The fluorescence of TO-positive iRBCs was measured in the FL_2_ channel. The reference negative control (blue curve histogram, Pool NEG), the positive control (green curve, Pool HIS), and a strong responder from Ndiop (red curve, positive sample) are shown. Analysis was done by Flow Jo® Software.

Membrane-bound IgG were revealed after a first incubation of 30 min at 37°C with plasma diluted 1 : 20, followed by a second incubation of 30 min at 37°C with phycoerythrin- (PE-) conjugated goat anti-human IgG diluted at 1 : 200 (Cappel, Organon Teknica, PA, USA). Live parasites were then labeled for 30 min at 37°C in the dark with thiazole orange (TO) (Retic-Count®; Becton-Dickinson, San Jose, CA, USA). Fluorescence was read within 1 h on a flow cytometer FACS with CellQuest® software (Becton-Dickinson®, San Jose, CA, USA). After gating on the TO-positive iRBC, 5,000 events for the overall iRBC (FL_1_ > 10^1^) were counted. At the end of the first set of analysis, gating of iRBC was changed to select mature forms of the parasites with subsequent nuclear material (FL_1_ > 10^2^), and 2,000 events were counted. In all cases, PE-labeled membrane-bound IgG was measured in the FL_2_ channel (Figure 1).

A pool of 30 plasma samples with high levels of IgG to whole schizont extracts, R23,* Pf*EB200, and/or* Pf*EMP3/5 [35], was used as the positive control (SHI), and pools of plasma obtained from non-*P. falciparum* exposed Europeans and Senegalese (Dakar inhabitants) were used as the negative controls. For the quantification of labeled iRBC, a labeling index (LI) ratio (LIR) was defined as LI_sample_/LI_negative control_. The LI was calculated as the percentage of iRBC with bound IgG (FL_1_ > 10^1^; FL_2_ > 10^2^) multiplied by the geometric mean intensity of the peak.

As previously described, this calculation allows interassay comparison [17] resulting in strong correlation between LIR and percentage of iRBC IgG binding. Analyses for flow cytometry were done with Flow Jo X 10.0.6 Software (Tree Star Inc., Ashland, OR, USA).

### 2.6. Statistical Analysis

As Ab responses are nonnormally distributed, comparisons of Ab levels and/or LIRs in different assays or groups were done using the Wilcoxon signed-rank test and the Spearman rank correlation test for paired data. The Mann-Whitney test was used to compare unpaired data. Comparison of prevalence for positive responders was done using the Chi^2^ test. *p* values of <0.05 were considered significant. Statistical analyses were performed with Statview®, version 5.0, software (SAS Institute, Cary, NJ, USA).

## 3. Results

### 3.1. Characteristics and Biological Data of Study Population

A total of 141 malaria patients were selected for the present study. The patients included 69 CM and 72 MM subjects. They suffered of confirmed clinical malaria symptoms within half a day to 14 days before the day of enrolment (mean 6 days).

The age of the patients ranged from 2 to 63 years (mean 17.6 years) without a significant difference between CM and MM groups that counted comparable numbers of adults (≥15 years) and young individuals (<15 years) (Table 1).

Regarding biological parameters, hemoglobin level and red blood cells count were significantly lower (*p* < 0.05) while the leucocytes count was significantly increased in CM compared to MM patients (*p* = 0.012). In contrast, there was no significant difference in the platelet count and hematocrit levels between the two groups. Interestingly, the parasitemia at the day of admission was not significantly different between the two groups of patients (Table 1).

There was no significant association between hematology parameters and parasitemia at inclusion in the CM group, contrary to the MM groups where hemoglobin level appeared negatively correlated to parasite densities (*r* = −0.53; *p* = 0.017). Furthermore, hematology data, except for platelet count, correlated together in all clinical groups.

According to age of individuals, that is, adults (≥15 years) versus young individuals (<15 years), no relationship was found between parasitemia and hemoglobin levels, red cells, white cells, or platelets count, in the two groups of patients.

In CM, no difference of parasitemia was detected between survivors and fatal cases (data not shown). In contrast, hemoglobin, hematocrit, and red blood cells count appeared lower in the fatal CM patients than in recovering individuals (*p* < 0.05). Hyperleucocytosis was significantly found in the group of patients with fatal outcome (*p* < 0.03).

### 3.2. Levels and Prevalence of IgG Responses in CM and MM Groups

A summary of antibodies levels and prevalence of responders is shown in Table 2. The number of positive responders against parasite extracts (whole schizont, 69% versus 50%; *p* = 0.021; iRBCm, 88% versus 50%; *p* < 0.01) was significantly higher in the MM compared to the CM group. In contrast, the prevalence of responders to the recombinant antigens did not significantly differ between the two groups.

Regarding levels of Ab responses, the mean OD ratios of the MM patients were higher than CMs for IgG to iRBCm (3.87 in MM and 2.75 in CM; *p* = 0.032) and to* Pf*EMP3/5 (3.82 in MM and 2.59 in CM; *p* = 0.031) (Table 2). In addition, IgG responses to iRBCm and to* Pf*EMP3/5 were positively correlated in CM (rho = 0.32; *p* = 0.029) and in MM patients (rho = 0.63; *p* < 0.001). Abs to other Ags were comparable between the two groups.

Regarding age of individuals, no significant difference of IgG Ab levels was found between adults (≥15 years) and children (<15 years) for all Ag tested in MM group (not shown). In CM patients, levels of IgG to iRBCm were positively correlated with the age of individuals (rho = 0.63; *p* = 0.001) and Ab levels were significantly higher in adults with CM than in CM children (*p* = 0.01).

### 3.3. Variations of IgG Antibodies Levels according to CM Outcome

**Figure 2:**
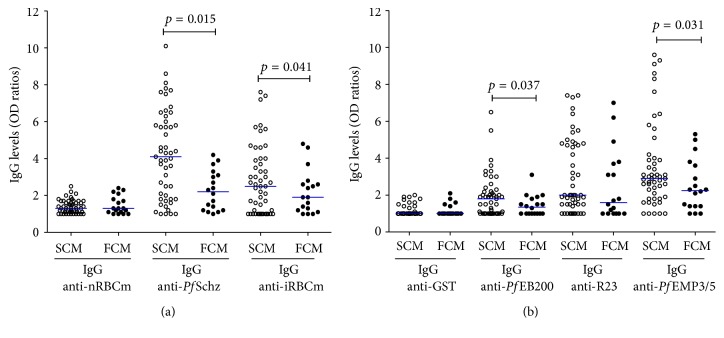
IgG antibodies levels against whole parasite extract antigens (a) and recombinants proteins (b) in survivors and fatal CM patients. Scatter plot of IgG responses to whole parasite extracts antigens (a) and recombinant proteins (b). Comparison between surviving cerebral malaria patients (SCM; *n* = 51; open circles) and fatal cerebral malaria patients (FCM; *n* = 18; black circles) for each antigen. Horizontal blue bar indicates medians of OD ratio value. The Mann-Whitney rank test was employed for between-groups comparison for each antigen. IgG levels were highest in surviving CM group for* P. falciparum* schizont extract, iRBC membrane,* Pf*EB200, and* Pf*EMP3/5. Significant *p* values are included (*p* < 0.05).

IgG response against selected Ag was compared between the survivors (SCM) and fatal cases of CM (Figure 2). SCM patients displayed significant higher levels of IgG to the crude extracts (Figure 2(a)) and to the recombinant proteins except for R23 (Figure 2(b)).

In patients with fatal outcomes (FCM), median levels of IgG anti-*Pf*Schz were twice lower: 2.2 versus 4.1, respectively (*p* = 0.015). A similar profile was observed for IgG anti-iRBCm with median values of 2.5 (25% (1st Quartile) = 1.0; 75% (3th Quartile) = 4) in SCM group versus 1.9 (25% (1st Quartile) = 1.2; 75% (3th Quartile) = 2.6) in FCM (*p* = 0.041) (Figure 2(a)).

### 3.4. Levels of IgG Binding Capacity according to the Severity and Outcome of the Disease

**Figure 3:**
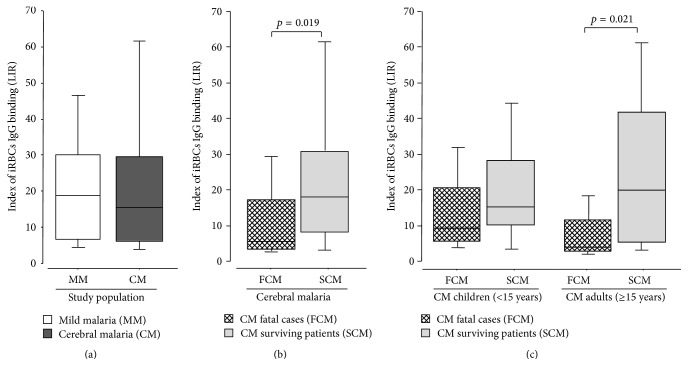
Variations of labeling index ratio according to disease severity (a), CM outcome (b), and age of individuals in CM patients (c). Box plot of labeling index ratio (LIR). The horizontal line within each box represents the median value of LIR. Comparison were done (a) between mild malaria patients (MM, *n* = 35; white boxes) and cerebral malaria group (CM, *n* = 69; dark boxes), (b) between fatal cerebral malaria group (FCM; *n* = 18; hacked boxes) and surviving cerebral malaria patients (SCM; *n* = 51; grey boxes), and (c) in CM patients according to both outcome and age of children (left) and adults (right). The Mann-Whitney rank test was employed for comparison between groups. Sera from survivors showed significant high LIR compared to fatal CM patients (*p* < 0.05) in adults. Significant *p* values are included.

Binding of live iRBCs measured by flow cytometry was analyzed in the context of the clinical status, disease outcome, and age of individuals. As shown in Figure 3(a), sera from MM patients showed a higher but not significant labeling index ratio (LIR) than the CM individuals.

We next compared the IgG binding to live iRBC from SCM versus FCM patients. The median LIR was significantly higher in the SCM compared to the FCM patients (Figure 3(b)). The index of iRBCs IgG binding was highly variable within the two groups, with median LIR significantly lower in fatal cases than in SCM group (6.40 ± 3.01 versus 19.40 ± 2.45;* p* = 0.019, resp.).

This result was partly available after age dichotomization of CM patients, although the difference did not reach significant level in children (Figure 3(c)). In CM surviving children, levels were slightly higher (16.00 ± 3.16) than in those who died (10.10 ± 3.70) (*p* = 0.207).

In adults, a significant (*p* = 0.021) higher level of iRBCs recognition was found in CM surviving (median = 20.70 ± 4.71) compared to fatal (median = 4.90 ± 3.03) cases.

More importantly, index of iRBCs IgG binding from surviving adults and children was not significantly different (*p* = 0.178). Only adults with fatal outcome showed significant lower LIR than children who deceased (*p* = 0.034).

No correlation was found between age of individuals and binding capacity of IgG to iRBCs either as continuous or as stratified variable (young, 2–8 years old; older children, 9–15 years; adults, ≥15 years). Additionally, no relation between parasitemia and LIR was observed in both MM and CM groups.

### 3.5. Relationship between iRBCs IgG Binding and Antibody Levels

The correlation between levels of IgG anti-*P. falciparum* and magnitude of iRBCs IgG binding was analyzed as function of disease severity, age of individuals, and clinical outcome.

**Figure 4:**
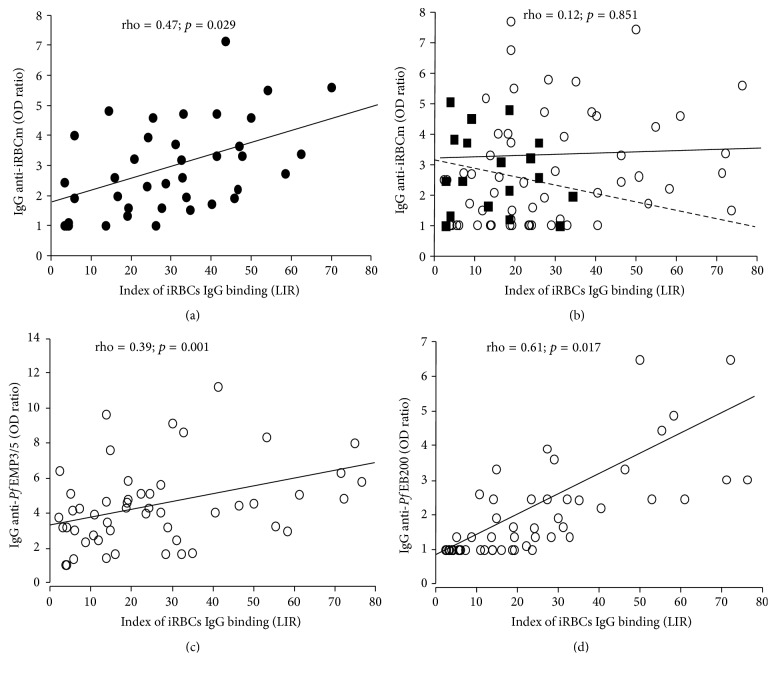
Relationship between labeled index of recognition and IgG responses in MM (a) and CM (b–d) groups. The correlations between labeled index of recognition (LIR) and IgG anti-iRBCm levels (OD ratio) are plotted in MM patients (a) (dark circles) and in CM group (b) with a dichotomization in fatal CM (dark squares) and surviving CM (open circles). Relationships between LIR and the levels of IgG anti-*Pf*EMP3/5 (OD ratio) or IgG anti-*Pf*EB200 levels (OD ratio) are, respectively, plotted in (c) and (d), only in CM surviving patients (open circles). Results from statistical analyses done by nonparametric Spearman rank test are indicated.

In MM group, index of serum IgG binding to iRBCs was positively correlated to IgG levels against infected erythrocyte membrane (iRBCm) (rho = 0.47; *p* = 0.029) (Figure 4(a)), IgG anti-*Pf*Schz (rho = 0.35; *p* = 0.037), and IgG anti-*Pf*EMP3/5 (rho = 0.53; *p* = 0.018). However, LIR had no significant relationship with age of MM patients.

Globally, in CM groups no correlation was found between LIR and levels of IgG anti-iRBCm (rho = 0.12; *p* = 0.851) or IgG anti-*Pf*Schz (rho = 0.06; *p* = 0.587). According to CM outcome, a trend for negative correlation was observed between levels of IgG anti-iRBCm and LIR in the group of fatal cases (rho = −0.16; *p* = 0.057) (Figure 4(b)). Furthermore, considering age and disease outcome, the previous trend was found in fatal CM adults but not in CM children.

For recombinant proteins, levels of IgG anti-*Pf*EMP3/5 (rho = 0.39; *p* = 0.001) (Figure 4(c)) and IgG anti-*Pf*EB200 (rho = 0.61; *p* = 0.017) (Figure 4(d)) were positively correlated to LIR in SCM group. When CM adults and children were analyzed separately with stratification on the outcome, the positive correlation were stronger in surviving CM adults (rho = 0.44; *p* < 0.001 for IgG anti-*Pf*EMP3/5 and rho = 0.72; *p* = 0.011 IgG anti-*Pf*EB200) than in CM children who recovered (rho = 0.29; *p* < 0.01 for IgG anti-*Pf*EMP3/5 and rho = 0.54; *p* = 0.031 IgG anti-*Pf*EB200) (Figure 4(b)).

## 4. Discussion

In this study, we analyzed Ab responses against* P. falciparum* total and recombinant Ags in individuals living in an urban area of low endemicity who suffered of acute symptoms of malaria requiring hospitalization. Two groups of patients (MM and CM), considered as nonimmune or partially immune and residing in the same locations, were selected and categorized on the basis of the disease severity. These individuals, regardless of age, of previous history of infection, and of individual conditions of exposition to infective bites, can be considered as being equally at risk for infection resulting in confirmed clinical outcomes with or without severe cerebral symptoms.

Antibodies are known to reduce morbidity and parasite densities in patients with malaria as demonstrated by passive transfer experiments [13, 36]. Intraerythrocytic circulating parasites synthesize a family of proteins displayed at the surface of the red blood cell. The proteins at the host-parasite interface play an important role in malaria pathogenesis: these antigens are under constant immune pressure and diversify to avoid immune detection while maintaining conserved features required for their function in host-parasite interactions. Antibody response against iRBCs surface proteins is a crucial step in initiating the adaptive immune response against the malaria infection as they can promote destruction and/or phagocytosis of iRBCs and subsequent parasite clearance. Analyzing how antibodies interact with iRBCs could be important in understanding the transition from innate to adaptive immunity and develop iRBCs-derived vaccine candidates and a reliable functional-associated monitoring technique of the IgG binding to iRBCs surface.

In this study, we aimed to evaluate the capacity of acquired IgG responses to bind live iRBCs with regard to potential relationship with defined iRBCs antigens and potential prognostic significance in symptomatic urban malaria. We used two whole parasite extracts and three recombinant proteins associated with the surface of iRBCs and shown to protect monkeys by eliciting strong opsonizing Ab responses [37].

The flow cytometry technique has been already used as a relevant surrogate marker in IgG binding to iRBC in different contexts: before and after vaccination trials [18], as function of age and hemoglobin type in Malian children [38] and as a differential marker in Senegalese individuals living in rural settings with different levels of endemicity [17]. In urban setting, previous studies focused on relationship between antibody responses to defined antigens and clinicoepidemiology such as age of individuals and severity of outcome [23, 33]. However, as reported in rural symptomatic malaria, the amount of sequestered parasites increased with disease severity [7], and the binding of* P. falciparum*-infected RBCs to microvascular endothelial cells induces pathological sequelae in microvessels that are associated with the symptoms and severe manifestations such as CM [39].

In this study, the iRBCs binding assay did not show any significant difference of LIR between CM and MM groups but, importantly, there was a significant lower IgG binding capacity to iRBCs in CM patients with fatal outcome compared to surviving group. These results are in agreement with the protection-associated role of IgG to iRBCs expressed antigens [40]. However, such significant difference between surviving versus fatal cases was observed here in adults (≥15 years); this may be attributed to the high anti-*P. falciparum* immune responses described in adults compared to children [8, 23]. To assess the potential predictive role of IgG binding capacity to iRBCs in severe malaria, more studies investigating this phenomenon are warranted, including IgG subclasses that can differentially promote several mechanisms [41] including immune phagocytosis [42].

When regarding technical aspects, the use of live parasite and flow cytometry supports substantial standardization [43] providing comparable interassays results in different studies [15, 17, 18]. Furthermore, the use of FCR3 strain of* P. falciparum* adapted to* in vitro* culture has been investigated; a number of changes in plasmatic membrane including a strong expression of knob-like structures involved in vascular adhesion and sequestration were described [44]. Indeed, the FCR3 strain used here has a limited relationship with natural parasites infecting population in urban Dakar; it may probably underestimate the actual individual IgG binding capacity. To circumvent the limited Ag repertoire expressed* in vitro* by FCR3 strain of* P. falciparum*, further analysis using local strains of parasites should be conducted. Implication of some other factors such as host genetic [38] and hemoglobin types [17] should be also investigated in urban setting.

For the determination of antibody responses, the use of whole parasite extract antigens and recombinants proteins showed that IgG responses to iRBCm and* Pf*EMP3/5 were positively correlated and their levels were significantly higher in MM compared to CM patients. These results are in agreement with previous findings of association with protection reported in* Saimiri* models [37] and in human [31]. This antigen, derived from the parasite-encoded protein* Pf*EMP3, is expressed in knobs structures and described as inducing a strong propensity for iRBCs to adhere in the vasculature [31]. However, protective role of Abs to* Pf*EMP3/5 and other iRBC expressed proteins such as* Pf*EB200 is difficult to establish for several reasons. Firstly, a large array of antigens expressed on the surface of infected erythrocytes [45] have been identified as potential targets of protective immunity and promising vaccine candidates [10]. Secondly, Bull et al. demonstrated that severe malaria resulted from a substantial limitation of the antibodies repertoire to variant antigens (VSAs) [11] and the main antigenic ligands responsible for both cytoadherence and antigenic variation are members of the* P. falciparum* Erythrocyte Membrane Protein-1 (*Pf*EMP1) family [44–46].

These iRBC surface proteins are highly diverse and undergo clonal antigenic variation under selective pressure by immune responses [47, 48]. They are dominantly recognized by antibodies from individuals with uncomplicated malaria [49], and the surface antigens of those parasites seem to be geographically conserved [50], supporting the hypothesis that the surface molecules expressed by parasites causing severe malaria, especially cerebral malaria, maintain relatively conserved epitopes. Indeed, the age-related acquisition of protection in endemic areas requires both strain-specific and strain-transcending IgG responses to iRBCs as reported by measuring anti-VSA binding assay in different settings of Tanzania [51]. In addition the strain-specific cumulative repertoire increases but was shown not to be infinite with a relative cross-geographical conservation [50]. It is clear that optimal anti-IRBCs Ab response would require additional anti-VSA measure, but VSA measure requires heavy simultaneous culture procedure of multiple strains which is more difficult than a potential use of one strain and/or defined Ag targets in the case of hospitalized patients with severe symptoms.

Thus, recombinant proteins tested in our study such* Pf*EMP3/5 and* Pf*EB200 are predominantly expressed in* P. falciparum* knobs structures [29, 30] whereas the presence of knobs may not necessarily result in sequestration. For example,* P. malariae* has knob structures but does not sequester, while* P. chabaudi* sequesters without knobs [52].

Taken together, our results underline the difficulty to establish a clear correlation between antibodies analyzed by ELISA and protection against disease outcome [40, 42, 53]. The use of ELISA alone cannot provide information about the function of antibodies [48]. The live iRBCs binding assay introduces a potential functional-associated assay for symptomatic malaria analysis. Here the absence of correlation of LIR with parasitaemia agrees with previous studies reporting measuring anti-erythrocyte surface antibodies [40] or iRBC binding capacity by agglutination [53] with protection from clinical malaria in endemic area. It is likely that these antibody responses are short-lived [54] and the presence of antibodies without parasites may simply reflect a recently treated acute infection, possibly a marker of increased susceptibility [40].

## 5. Conclusion

ELISA provides distinct measure of the anti-*P. falciparum* IRBC responses compared to iRBCs binding assay. Our results showed a significant difference of LIR between surviving and fatal outcome in patients with confirmed cerebral malaria. This IgG binding assay has potency for analysis of symptomatic malaria; it deserves further investigation to determine to what extent it could provide a relevant indicator highlighting a risk of fatality in cerebral malaria.

## Figures and Tables

**Table 1:** Epidemiological and hematoparasitological characteristics of the study population.

Parameters	Cerebral malaria (*n* = 69) Mean ± SE [min–max]	Mild malaria (*n* = 72) Mean ± SE [min–max]	*p*
Gender (male/female)	39/30	43/29	*—*
Age (year)	17.9 ± 1.8 [2–63]	17.3 ± 1.2 [5–55]	ns
Age groups (adults/children)	39/30	35/37	*—*
Parasitemia (Tr/*µ*L)	30150 ± 5510 [175–452100]	28348 ± 3755 [220–141000]	ns
Haemoglobin (g/dL)	8.4 ± 0.33 [3.3–15.1]	11.5 ± 0.51 [8.3–16.1]	*0.031*
Red blood cells count (×10^6^/*µ*L)	3.14 ± 0.13 [1.18–4.98]	4.72 ± 0.18 [2.90–7.71]	*0.040*
White blood cells count (×10^3^/*µ*L)	12.84 ± 1.15 [4.90–51.10]	8.21 ± 1.01 [4.5–13.3]	*0.012*
Platelet count (×10^3^/*µ*L)	143.4 ± 16.2 [11–533]	168.2 ± 11.8 [28–355]	ns
Hematocrit (%)	31.1 ± 0.22 [10.5–46.1]	35.9 ± 0.18 [23.9–49.1]	ns

SE = standard error, Min = minimum, Max = maximum, ns = nonsignificant, and *n* = number of patients. Data were determined by the hospital's medical laboratory. Adults were patients ≥15 years and children were patients <15 years. *p* = *p* value of comparison between CM and MM groups with Mann-Whitney rank test.

**Table 2:** Levels and prevalence of anti-IgG responses against tested antigens.

Parameters		Cerebral malaria (*n* = 69)	Mild malaria (*n* = 72)	*p*
IgG anti-*Pf*Schz	*N* (%)	35 (50)	50 (69)	*0.021*
	ODrt ± SE^*∗*^	3.80 ± 0.29	3.06 ± 0.42	*0.063*

IgG anti-iRBCm	*N* (%)	35 (50)	63 (88)	<*0.01*
	ODrt ± SE	2.75 ± 0.23	3.87 ± 0.39	*0.032*

IgG anti-*Pf*EB200	*N* (%)	15 (22)	19 (26)	*0.518*
	ODrt ± SE	1.38 ± 0.14	1.21 ± 0.36	*0.235*

IgG anti-R23	*N* (%)	10 (14)	16 (22)	*0.236*
	ODrt ± SE	2.27 ± 0.33	1.98 ± 0.31	*0.452*

IgG anti-*Pf*EMP3/5	*N* (%)	37 (54)	30 (42)	*0.155*
	ODrt ± SE	2.59 ± 0.54	3.82 ± 0.38	*0.031*

^*∗*^IgG responses in OD ratio ± standard error.

*N* = number of positive responders that is individuals with OD ratio ≥2, (%) = prevalence of positive responses, ODrt = Mean Optical Densities ratio, SE = standard error, *p* = *p* value resulting from comparison with Mann-Whitney rank or Chi^2^ tests employed, respectively, for ODrt and prevalences of responders.
